# Fluorescent nanodiamonds as a relevant tag for the assessment of alum adjuvant particle biodisposition

**DOI:** 10.1186/s12916-015-0388-2

**Published:** 2015-06-17

**Authors:** Housam Eidi, Marie-Odile David, Guillemette Crépeaux, Laetitia Henry, Vandana Joshi, Marie-Hélène Berger, Mohamed Sennour, Josette Cadusseau, Romain K. Gherardi, Patrick A. Curmi

**Affiliations:** Institut National de la Santé et de la Recherche Médicale (INSERM) - UMR 1204, Université Evry-Val d’Essonne, Laboratoire Structure-Activité des Biomolécules Normales et Pathologiques, Evry, France; Laboratoire Pierre-Marie Fourt, Centre des Matériaux de l’Ecole des Mines de Paris and CNRS UMR 7633, Evry, France; Inserm - U955, Université Paris Est, Faculté de Médecine, Créteil, France; Faculté des Sciences et Technologie UPEC, Créteil, France

**Keywords:** Alum, fluorescent nanodiamonds, vaccine adjuvant, biodisposition

## Abstract

**Background:**

Aluminum oxyhydroxide (alum) is a crystalline compound widely used as an immunologic adjuvant of vaccines. Concerns linked to alum particles have emerged following recognition of their causative role in the so-called macrophagic myofasciitis (MMF) lesion in patients with myalgic encephalomyelitis, revealing an unexpectedly long-lasting biopersistence of alum within immune cells and a fundamental misconception of its biodisposition. Evidence that aluminum-coated particles phagocytozed in the injected muscle and its draining lymph nodes can disseminate within phagocytes throughout the body and slowly accumulate in the brain further suggested that alum safety should be evaluated in the long term. However, lack of specific staining makes difficult the assessment of low quantities of *bona fide* alum adjuvant particles in tissues.

**Methods:**

We explored the feasibility of using fluorescent functionalized nanodiamonds (mfNDs) as a permanent label of alum (Alhydrogel^®^). mfNDs have a specific and perfectly photostable fluorescence based on the presence within the diamond lattice of nitrogen-vacancy centers (NV centers). As the NV center does not bleach, it allows the microspectrometric detection of mfNDs at very low levels and in the long-term. We thus developed fluorescent nanodiamonds functionalized by hyperbranched polyglycerol (mfNDs) allowing good coupling and stability of alum:mfNDs (AluDia) complexes. Specificities of AluDia complexes were comparable to the whole reference vaccine (anti-hepatitis B vaccine) in terms of particle size and zeta potential.

**Results:**

*In vivo*, AluDia injection was followed by prompt phagocytosis and AluDia particles remained easily detectable by the specific signal of the fND particles in the injected muscle, draining lymph nodes, spleen, liver and brain. *In vitro*, mfNDs had low toxicity on THP-1 cells and AluDia showed cell toxicity similar to alum alone. Expectedly, AluDia elicited autophagy, and allowed highly specific detection of small amounts of alum in autophagosomes.

**Conclusions:**

The fluorescent nanodiamond technology is able to overcome the limitations of previously used organic fluorophores, thus appearing as a choice methodology for studying distribution, persistence and long-term neurotoxicity of alum adjuvants and beyond of other types of nanoparticles.

## Background

The understanding of how the body handles small particles in the long-term, especially those which interact with the immune system, is a major objective of recent research [1]. For example, concerns linked to the use of aluminum particles as vaccine adjuvants [aluminum oxyhydroxide (“alum”)] have emerged following recognition of their role at the origin of the focal lesion called macrophagic myofasciitis (MMF). This revealed a fundamental misconception of the fate of alum in the organism pointing out its unexpectedly long-lasting biopersistence within immune cells [2]. It also demonstrated their capacity to migrate to the lymphoid organs, to disseminate throughout the body within monocyte-lineage cells, and to slowly accumulate in the brain [3]. Millions of humans have received vaccines adjuvanted with alum. Overall safety of these vaccines has been regarded as excellent at the level of the population [4], but adverse effects have also been reported [5, 6]. It seems very likely that a small proportion of presumably susceptible individuals exposed to particulate materials with adjuvant effects, e.g. alum adjuvants or breast implant-derived silicone, may develop progressive systemic and neurologic autoimmune/inflammatory manifestations or “ASIA” [7]. These individuals typically show long-term persistence of particles within the monocyte-lineage cells at either the site of previous immunization with alum-containing vaccines, i.e. MMF, or in the vicinity of leaky breast implants [8].

Alum particles have neither fluorescent nor magnetic properties. Their detection in tissues therefore represents a difficult challenge. Khan *et al.* [3] analyzed biodisposition of alum particles in mice by tracking fluorescent alum surrogates, such as alum-like hybrids which were composed of a rhodamine core coated with precipitated aluminum hydroxide. This approach has limitations since the precipitated aluminum hydroxide used by Khan *et al.* [3] is similar but not strictly identical to the aluminum oxyhydroxide used in vaccines [9]. Indeed, particles may exhibit strikingly different properties according to their physicochemical properties, the main parameters being their size, shape, zeta potential and chemical composition [10].

The present study aimed at evaluating the possibility of constructing a fluorescent complex highly relevant to vaccine by tagging the alum adjuvant itself (Alhydrogel^®^) using modified fluorescent nanodiamonds (mfNDs). MfNDs have unique fluorescence properties, which allow their detection at very low levels and over a very long-term period [11–13]. Indeed, their fluorescence, based on the presence of nitrogen-vacancy centers (NV centers) within the nanodiamond crystal lattice, is perfectly photostable with neither bleaching nor blinking. mfNDs were reported as biocompatible fluorescent particles with very low toxicity [14]. These properties overcome the limitations of organic fluorophores or quantum dots, i.e. photobleaching and toxicity [15–17]. In our last paper we showed that fNDs functionalized with hyperbranched polyglycerol (mfNDs) could be promising tools for biomedical research [18].

In the present study, Alhydrogel^®^ used in vaccines was tagged with mfNDs forming the AluDia complex. We first determined, under different conditions, AluDia physicochemical properties, including morphology, size, zeta potential and stability. Then, we examined the fate of AluDia after injection into mouse muscle in terms of granuloma formation and persistence, and biodistribution to distant organs. Finally, we analyzed the effects of AluDia administration to macrophages and neurons in culture, including cytotoxicity, internalization, stability and intracellular behavior.

## Methods

### Preparation and characterization of the AluDia complex

Reagent grade chemicals were purchased from Sigma–Aldrich (France) and used as received. The suspension of aluminum oxyhydroxyde or alum (Alhydrogel^®^) 2 wt % in water was purchased from Invivogen. Commercially available anti-hepatitis B vaccine (ENGERIX B^®^, GlaxoSmithKline Inc., Evreux, France) was used as a reference for its physico-chemical specificities.

#### Fluorescent nanodiamonds preparation

Fluorescent nanodiamonds (fNDs) were prepared from a synthetic micron diamond powder (Element Six PDA999 80–100 mesh) as already described [13]. The creation of NV centers in the crystal, the origin of fluorescence, was done by electronic irradiation followed by annealing at 850 °C under vacuum. To convert the micron size to nano size powder, nitrogen jet milling followed by planetary ball milling was used. Then fNDs were extracted, decontaminated by treatment with harsh acids (HF/HNO_3_) and washed with water. An additional treatment with perchloric acid was used to improve cleaning and saturate the surface with oxygenated chemical groups [18].

#### Synthesis of modified fluorescent nanodiamonds

A total of 5 mg fNDs powder was dispersed in 3 mL hyperbranched polyglycerol (HPG) and the reaction carried out at 140 °C for two hours as described by Boudou *et al.* [18]. The mfND sample was isolated from the reaction medium by dissolution of the free polymer and residual glycidol in methanol and subsequent centrifugation at 15,000 x g for 30 minutes. This procedure was repeated three times. The final pellet was further washed by three repeated dispersions in water followed by centrifugation at 15,000 x g for 30 minutes. The final mfND pellet was dispersed in water for further use.

#### MfND-Alhydrogel^***®***^ complex formation (AluDia complex)

Before use, the suspensions of mfNDs (1.3 g/L) and alum (10 g/L) in water were sonicated for five minutes. The complex (AluDia) was obtained by mixing the Alhydrogel^®^ and mfND suspensions at a ratio of 1:17 v/v followed by a thorough agitation and a few minutes of sonication. The AluDia suspension was then diluted to reach the appropriate concentration in PBS.

#### Size and zeta potential measurements

We characterized the size distribution and zeta potential of mfNDs, alum and AluDia as well as of the particles present in a commercially available anti-hepatitis B vaccine (HBV, ENGERIX B^®^ GlaxoSmithKline Inc., Evreux, France) using dynamic light scattering. Nanoparticles/agglomerates were suspended in water, to a concentration <1 mg/mL. pH/ conductivity was controlled by an autotitrator Delsa™Nano AT, and the suspension was analyzed using a Delsa Nano C Particle Size apparatus, (Beckman Coulter, Villepinte, France) equipped with two 658 nm laser diode sources (power 30 mW) and a temperature controller (from 15 to 90 °C). Size distribution was approximated by photon correlation spectroscopy (PCS, also called dynamic light scattering) at a scattering angle of 165°. Scattering data were collected for 70 individual measurements at a constant scattering angle and averaged for each sample. When possible, the particle zeta potential was determined by measuring the electrophoretic movement of the charged particles under an applied electric field. Scattered light was detected at a 15° angle and a temperature of 25 °C.

### *In vitro* and *in vivo* experiments

#### Cell culture

We used THP1 and NSC-34 as *in vitro* models. These cell lines were used to assess particle internalization, intracellular colocalization of alum and mfNDs particles and their cytotoxicity.

The human THP-1 monocyte cell line was grown in RPMI-1640 medium supplemented with 10 % fetal bovine serum, 2 mM L-glutamine, 100 U/mL penicillin, and 100 mg/mL streptomycin, under standard conditions (humidified chamber at 37 °C and 5 % CO2 atmosphere).

The murine NSC-34 cells, a hybrid cell line consisting of motor neurons fused with neuroblastomas, were grown in D-MEM culture medium (Fisher Scientific, Illkirch, France), complemented with 10 % heat-inactivated fetal bovine serum (Gibco BRL, Illkirch, France), 50 U/mL penicillin, and 50 mg/mL streptomycin. Cells were incubated in a humidified chamber at 37 °C and 5 % CO2 atmosphere. THP-1 and NSC-34 were grown in suspension and in two-dimensional flasks, respectively.

#### Evaluation of cell viability by MTT assay

To assess cell viability, cells were seeded in 96-well plates at a density of 1.3 x 10^3^ cells/well in 100 μL of medium. Particle toxicity in cells was assessed using the 3-(4, 5- dimethylthiazol-2yl)-2,5-diphenyltetrazolium bromide, or MTT, cell viability assay. Cells were treated with different doses of alum, mfNDs and AluDia particles two days after the beginning of the culture. The viabilities of cultured cells were assessed 72 hours after treatment with mfNDs, alum and AluDia particles following the manufacturer’s protocol. Briefly, cells were incubated at 37 °C with 10 μL/well of MTT solution for 70 minutes. After a 3-hour incubation at 37 °C under 5 %CO2, the MTT solution was discarded carefully and the blue formazan crystals, formed by reduction of MTT, were dissolved in dimethyl sulfoxide (DMSO [67-68-5], Sigma–Aldrich). The amount of formazan was determined spectrophotometrically by measuring the absorbance at λ = 540 nm using a microplate reader (Model 3550-UV, Bio-Rad, Marnes-la-Coquette, France). Each concentration was tested in quadruplicate and three independent experiments were performed. Since absorbance is proportional to the number of living cells, cell viability was represented by the absorbance ratio of exposed to control cells.

Since incubation with aluminum adjuvant was previously shown to increase the mitochondrial activity [19] which might interfere with MTT results, cell toxicity was confirmed by the Trypan blue test (data not shown).

#### Mice model

All animal experiments were conducted in accordance with the European guidelines for animal care. Eight male 8- to 10-week‐old C57BL/6 mice with an average weight of 25 g were used. Sixteen female 7-week-old CD1 mice were used to observe the granuloma size in injected muscles with AluDia particles at 45, 135, 180 and 270 days post injection (four mice per time). Mice were protected from Al‐containing materials, fed with manufactured animal food and water *ad libitum*, and exposed to 12:12 light/dark cycles.

#### AluDia administration

The dose of AluDia administered to mice was calibrated to mimic the mean number of human adult doses of the ENGERIX B^®^ vaccine received by MMF patients. A dose of 20 μL AluDia, corresponding to 400 μg Al/kg, was injected in the tibialis muscle of mice.

#### Tissue preparation and particle counting

On days 7 and 21 post-injection mice were transcardially perfused with PBS under terminal anesthesia. Tissues and organs were removed and quickly frozen. Whole brains were serially cut into 40 μm coronal cryosections, spleen and muscle into 20 μm, and draining lymph nodes (DLNs) into 12 μm, then stored at −20 °C until particle counting or treatment. Tissue sections were successively deposited on 10 different Superfrost^®^-plus slides in order to obtain 10 identical series, thus allowing determination of total particle content by multiplying by 10 the number of particles found in a single series.

#### Immunohistochemistry and Morin staining

CD11b labeling was used to observe the monocyte-lineage cells, including macrophages in injected muscles. Tissue sections were fixed by 2 % paraformaldehyde (PFA) and rinsed three times with PBS before using the immunodetection Kit (M.O.M™ kit, Vector^®^, Peterborough, UK). Anti-CD11b (MAC-1) (AbD SEROTEC - product code: MCA74GA, Oxford, UK) was used as “rat anti-mouse” primary antibody (1/200). Rat IgG produced in the donkey and coupled to Alexa Fluor 488^®^ fluorochrome (Jackson ImmunoResearch, Suffolk, UK) was used as the secondary antibody. After rinsing with PBS, the slides were coverslipped using Vectashield^®^ aqueous medium (Eurobio, Courtabœuf, France). Aluminum was stained after tissue section with Morin (M4008‐2G, Sigma‐Aldrich) that was dissolved in a solution consisting of 0.5 % acetic acid in 85 % ethanol [20]. Formation of a fluorescent Morin complex with aluminum was detected by the presence of an intense green fluorescence with a characteristic 520 nm emission under a 420 nm excitation.

#### Epifluorescence microscopy and microspectrometry

Fluorescence observations were made using a Zeiss Axioplan 2 microscope, equipped with a 1.4 NA oil immersion objective. Fluorescence images were obtained with a Princeton Instruments EMCCD Camera Rolera em–c^2^, with typical exposure times. For mfNDs detection, a DPSSL 532 nm (200 mW) laser beam was used as the illuminating source and was guided to the microscope by an optical fiber. A long pass 600 nm emission filter was used to collect only wavelengths higher than 600 nm. Spectra of the fluorescent spots were acquired by focusing the fluorescent object emission from the microscope onto an Acton SP2150i spectrometer (Princeton Instruments, Buckinghamshire, UK), and detected with a PIXIS–100B– eXcelon CCD camera (Princeton Instruments).

#### Electron microscopy

Cells were seeded for 24 hours in standard conditions (conditions similar to those used for fluorescence experiments). AluDia particles were added to the cell culture medium and incubated for 24 hours at 37 °C. Cells were then fixed in 4 % glutaraldehyde phosphate buffer for 45 minutes at room temperature. After dehydration with a graded series of ethanol, the cells were embedded in Epon resin. Ultrathin sections of the resin block were then cut (100 nm thickness) and stained with 2 % uranyl acetate for a higher contrast imaging. Electron microscopy observations were done with a high-resolution transmission electron microscope (HR-TEM-STEM, Tecnai F20ST operating at 200 keV with a field emission gun).

Energy-dispersive X-ray (EDX) analysis coupled with STEM was used to identify the elemental composition of AluDia complex. High resolution images were recorded at approximately Scherzer defocus on a CCD multiscan camera after astigmatism corrections, and eventually filtered via the Digital Micrograph software.

#### Statistical analyses

One-way ANOVA with Fisher’s Least Significant Difference (LSD) post hoc comparisons at 95 % confidence interval was used for statistical comparisons in the MTT assay. All statistical analyses were performed with the SigmaStat 3.11 software package (Sistat Inc., USA).

## Results and discussion

### Particle characterization

**Fig. 1:**
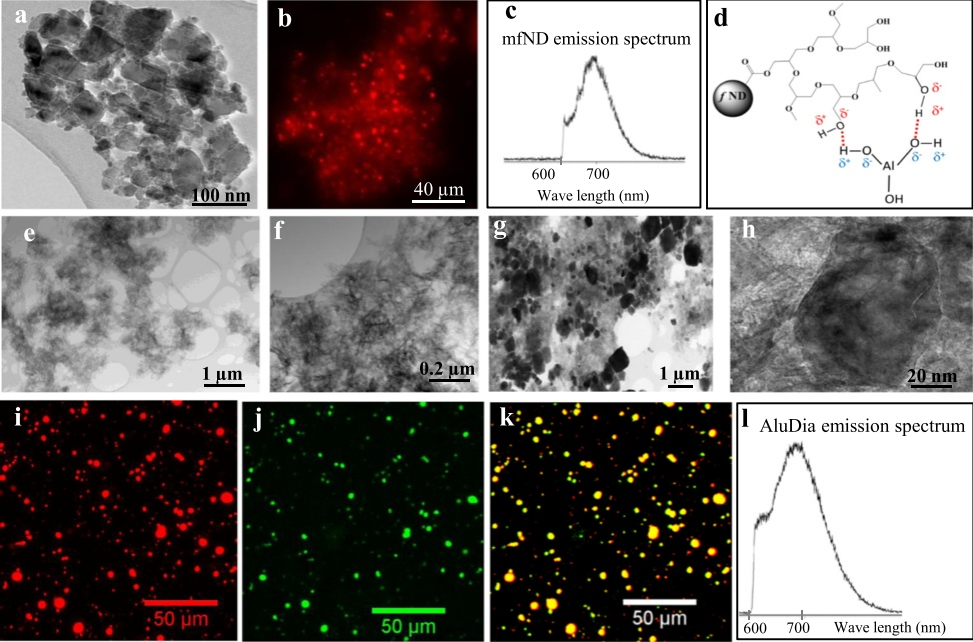
Particle characterization by microscopy. **a**: TEM analysis of fND aggregates that contained very small mfNDs (few nm). **b**: The red specific fluorescence of mfNDs excited by a 532 nm laser source. **c**: mfNDs luminescence spectrum with a specific peak at 700 nm. **d**: Schematic representation of the possible hydrogen bonds between the hydroxyl groups of the HPG chains and that of alum. **e** and **f**: The nanofibrous morphology of alum and vaccine aggregates (ENGERIX B^®^) by TEM analysis. **g** and **h**: The AluDia complex analyzed by TEM in which alum keeps its nanofibrous morphology and is loaded by non-fibrous, electron dense mfNDs. **i-k**: Fluorescence microscopy observation of an AluDia agglomerate. Red fluorescence of AluDia particles excited by a 532 nm laser source (**i**). Alum of AluDia complex stained with Morin and detected by a green fluorescence with a characteristic 520 nm emission when excited at 420nm (**j**), colocalization of the red fluorescence of mfNDs and the green Morin fluorescence of alum (**k**), without alteration of the typical mfND emission spectrum (**l**). *AluDia* Alhydrogel^®^ and mfND complex, *HPG* hyperbranched polyglycerol, *mfNDs* modified fluorescent nanodiamonds, *TEM* transmission electron microscope

As assessed by dynamic light scattering of particles in PBS at pH7.2, mfNDs appeared to be of nanometric size (about 80 nm) whereas agglomerates of alum alone, AluDia and Engerix^®^ alum formed particles of micrometric diameter with peaks from 2,900 nm to 3,800 nm (Table 1 and Fig. 1). Our characterization data showed that the zeta potential of mfNDs is slightly negative (−29 mV) whereas those of alum, AluDia, and ENGERIX^®^ particles are slightly positive, ranging from +25 to +30 mV (Table 1). Thus, in the physiological conditions we used, AluDia particle size and zeta potential were very similar to those of alum alone or alum adsorbed with HBV antigen. In addition, the size and charge of the AluDia particles remained stable during 15 days in PBS as well as in DMEM culture medium supplemented with 5 % (v/v) fetal bovine serum at 4 °C (data non shown). Thus, the physico-chemical properties of the AluDia complex were as close as possible to those of the HBV vaccine, making mfNDs relevant for further investigation as a tag for alum particle tracking.

**Table 1:** Diameter distribution and zeta potential values of mfND, Alhydrogel^®^, AluDia and ENGERIX^®^ alum particles

Particle types	Diameter (nm)	Zeta potential (mV)
mfNDs	80 ± 30	- 29 ± 3
Alhydrogel^®^	3240 ± 200	+25 ± 4
AluDia	2930 ± 230	+28 ± 2
ENGERIX^®^ alum	3820 ± 570	+30 ± 5

Size and zeta potential data were obtained in PBS at pH7.2 and 25 °C. Three different samples of each particle preparation were used in size and zeta potential analyses (n = 3 per particle type). Size and zeta potential were measured in triplicate in each sample (triplicate analyses)

The Alhydrogel^®^ and mfND ratio in the AluDia complex was determined on the basis of different experimental assays. The respective proportions of the suspensions Alhydrogel^®^ and mfNDs were varied from 1/0.25 to 1/100 and the resulting complex observed with fluorescent microscopy and characterized by TEM. The final value of 1/17 was chosen as the optimal compromise between these two distant values. With the 1/0.25 ratio, mfNDs were difficult to detect in the large amount of Alhydrogel^®^ during TEM observations. At the inverse, with the 1/100 ratio, Alhydrogel^®^ became almost the minor component. The preparations of the AluDia complex were based on the simple mixing of both solutions and their agitation to favor the complex formation. The formed particles had a micrometric size and they fell down rapidly to the bottom of the eppendorf. However, free mfNDs gave stable suspensions and should then remain in the supernatant. The analysis of the latter in the mixture case used in this study showed no free mfNDs.

HR-TEM imaging showed that mfNDs have a rounded to polygonal shape (Fig. 1a). In addition to the mfND size indicated by dynamic light scattering analysis, very small mfNDs (a few nm) were detected within the mfND aggregates by TEM. When excited by a laser at 532 nm, mfNDs displayed a red fluorescence (Fig. 1b) with a specific luminescence spectrum peaking at 700 nm (Fig. 1c).

We attribute the favorable interactions created between alum and mfNDs to the presence of numerous hydroxyl groups on the polyglycerol chains synthesized at the surface of mfNDs. Polyols were reported to be adsorbed strongly at the surface of boehmite (AlOOH) particles and hydrogen bonds were created between their hydroxyl groups and AlOOH [21]. Thanks to the hyperbranched structure of polyglycerol, numerous hydroxyl groups are present, along and at the end of the HPG chains, and can thus create such hydrogen interactions with alum (Fig. 1d). Although electrostatic interactions due to opposite zeta potential values between mfNDs and Alhydrogel^®^ cannot be completely excluded, the contribution of this second type of interaction to the stability of the complex should almost disappear in the salty solutions (PBS 1x) that we use due to the charge screening effect.

Both alum and vaccine particles displayed a nano-fibrous morphology (Fig. 1e, f), as previously reported in the literature [22]. AluDia were distinctly composed of nano-fibrous agglomerates of alum randomly decorated by polygonal, non-fibrous, electron dense mfNDs (Fig. 1g, h). This was confirmed by X-ray microanalysis and high resolution transmission electron microscopy (data not shown, see below). In bright field TEM images, the mfNDs have various crystal orientations with respect to the incident electron beam. The darker particles correspond to nanocrystals in Bragg’s position on which the electron beam has been diffracted and then stopped by the objective aperture (Fig. 1a, g and h). AluDia particles stained by Morin to detect aluminum showed colocalization of the red fluorescence of mfNDs with the green fluorescence of the Morin-alum complex, without alteration of the typical mfND emission spectrum (Fig. 1i-l). Thus, the association of mfNDs with alum does not disturb the fluorescent signature of mfNDs. Taken together, these results and the persistent colocalization observed at distant places from the injection point in mice (as will be shown later) strongly suggest that stable attractive interactions are created between aluminum oxyhydroxide and mfNDs. Moreover, fluorescence images (Fig. 1i-k) show clearly that mfNDs are much more fluorescent than alum stained by Morin which makes these particles useful to detect isolated alum particles.

Morin stain was previously used in the paper from our lab showing systemic translocation of Al particles [3]. However, Morin stain was reported to be not entirely specific for Al. Browne *et al.* showed that Morin could stain other metals but Al has the most of affinity to Morin according to the following order: Al > Fe(III) > Cu > Fe(II) > Ca > Mg > Mn = Zn [23]. Lumogallion was reported as an interesting dye for aluminum studies [24, 25]; however, it has limitations, too. Lumogallion specificity for Al is higher than that of Morin but Lumogallion also stains gallium, in addition to Al [26] and other metals, such as Fe [27, 28]. Furthermore, both the Lumogallion affinity for Al and fluorescence signal intensity are much lower than those of Morin [29] limiting its use when the detection of trace amounts of Al in tissues is concerned, e.g. in brain. Additionally, the Lumogallion red/orange fluorescence emission spectrum is very close to that of the mfNDs we used, precluding its use in place of Morin in the present study.

### *In vivo* observations

#### Granuloma formation at the injection site

**Fig. 2:**
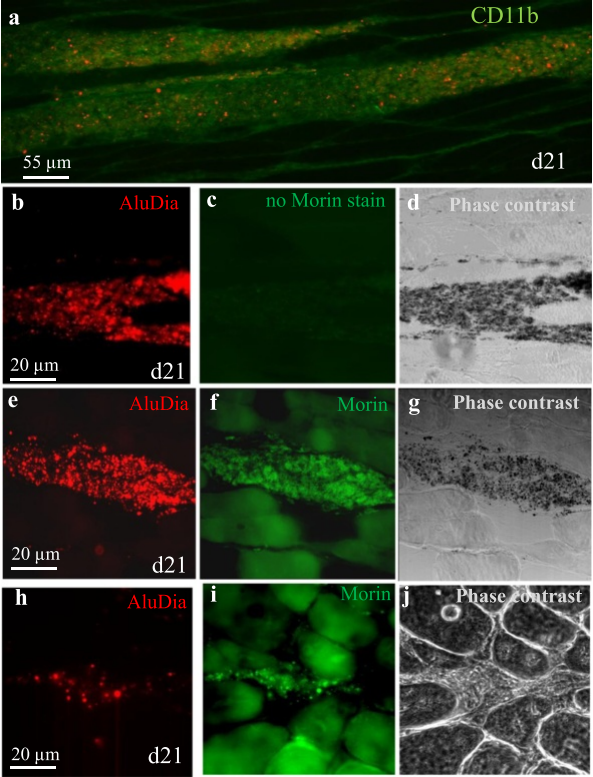
AluDia translocation and biopersistence in injected muscle after 21 days. **a**: immunohistochemistry analysis: AluDia particle accumulation in the injected muscle inducing a granuloma of CD11b+ monocyte-macrophage lineage cells filled with AluDia in the endomysium area. The green fluorescence corresponds to CD11b protein immunolabeling. b-d: AluDia particle detection (**b**) in injected muscle non-stained with Morin. Tissue section was observed using the 420 nm excitation for Morin (**c**) and phase contrast (**d**). **e-g**: the stable association between the mfND component as assessed by the red fluorescence obtained with the 532 nm laser excitation (**e**) and alum after Morin staining observed with 420 nm excitation (**f**) and phase contrast image (**g**). **h**: mfND component of AluDia complex (red fluorescence) with alum component stained with Morin and disturbed by the high fluorescent background of muscle cells (**i**) at the injection site in muscle section observed with phase contrast microscopy analysis (**j**). *AluDia* Alhydrogel^®^ and mfND complex, *mfNDs* modified fluorescent nanodiamonds

At 21 days after i.m. injection, AluDia particles accumulated into the injected muscle similarly to vaccine particles [3]. Indeed, granuloma mainly composed of CD11b+ monocyte- macrophage lineage cells filled with AluDia was formed in the endomysium, i.e. in between myofibers, at the injection site (Fig. 2a). Non Morin-stained AluDia particles in muscles have the same fluorescent signature as compared to those of mfNDs (Fig. 2b,c). These particles do not display any fluorescence when they are excited at 420nm as compared to Morin-stained AluDia (Fig. 2c). The phase contrast image shows AluDia particles within the granuloma region in muscle section (Fig. 2d). Morin stain for aluminum confirmed that macrophages contained stably associated AluDia particles as assessed by both red and green fluorescence (Fig. 2e-g). Importantly, photostability of mfNDs upon long laser exposure made AluDia detection very easy without background fluorescence whereas the detection of Morin stain was commonly disturbed by its bleaching and a strong tissue fluorescent background (Fig. 2h-j). Serial sectioning of the injected muscle at day 45, day 135, day 180 and day 270 after AluDia injection showed progressive shrinkage of muscle granulomas (Table 2), as previously reported in rats [30]. At 270 days post-injection, one out of three tested mice was completely free of muscle granuloma, and the other two mice only had small residual muscle granulomas.

**Table 2:** Semi-quantitative study of the progressive decrease of granuloma size in injected muscle with AluDia complex. *AluDia* Alhydrogel^®^ and mfND complex, *TA* tibialis anterior

Days	No granuloma (0)	Small granuloma (+)	Medium granuloma (++)	Large granuloma (+++)	Total
D45	2 %	13 %	24 %	61 %	100 %
D135	2 %	14 %	31 %	54 %	100 %
D180	36 %	22 %	9 %	19 %	100 %
D270	71 %	19 %	10 %	0 %	100 %

Whole TA muscle from three injected animals per time point (n = 3 per time point) were serially cryo-sectionned (a mean number of 90 longitudinal muscle sections were obtained per animal). Each section was categorized into four groups: no granuloma (0), small (+), medium (++) and large (+++) granuloma. Then, percentage of muscle section typing was calculated at each time point

#### AluDia translocation from the injected muscle to distant organs

**Fig. 3:**
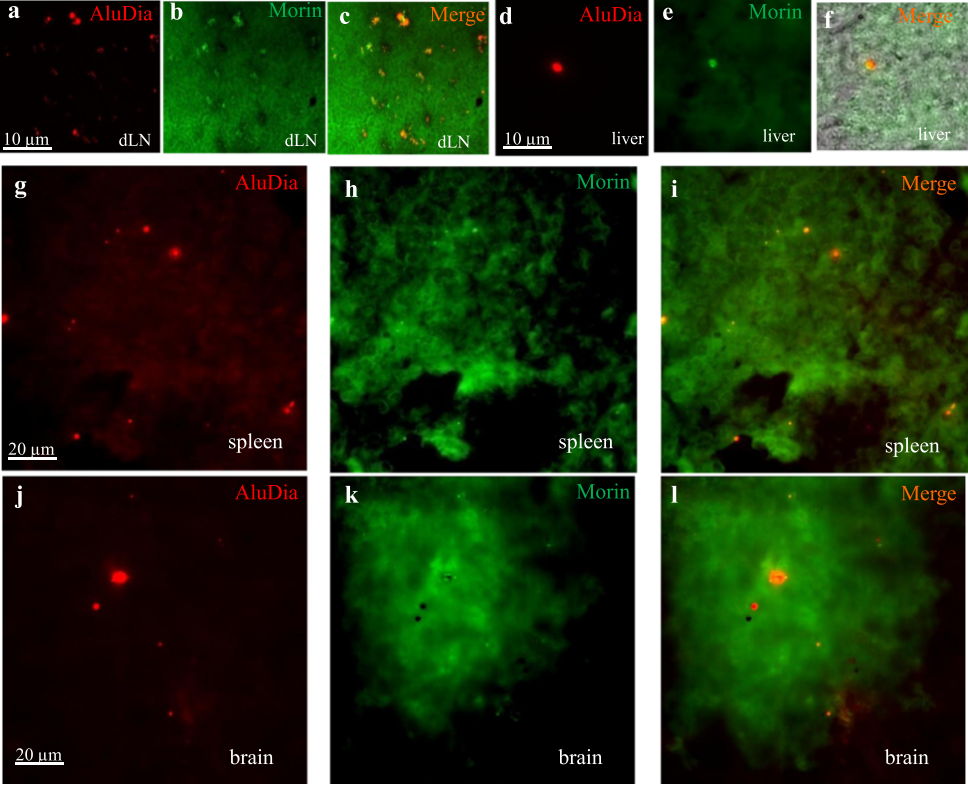
Assessment of AluDia biodistribution following its injection in tibialis anterior muscle at day 21. **a-c**: AluDia translocation in inguinal lymphatic nodes which appeared mostly empty at day 21 (cf. Table 2). **d-l**: AluDia particles reach liver, spleen and brain forming small clusters. In all observations, Morin stain of aluminum revealed that mfNDs and alum were co-localized in most particles. *AluDia* Alhydrogel^®^ and mfND complex, *mfNDs* modified fluorescent nanodiamonds

AluDia injected in the mouse tibialis anterior muscle was followed by lymphatic and systemic particle biodistribution (Table 3), as previously reported with other fluorescent particles [3]. Alum and mfNDs remained clearly colocalized in a large majority of particles detected remote from the injection site as assessed by Morin stain (Fig. 3a-i). Actually, our data of particle counting in sections from various tissues showed that 88 ± 4 % of observed nanodiamonds were close to those of alum. Similarly to alum-rhodamine nanohybrid particles (AlRho) used by Khan *et al.* [3], AluDia reached the inguinal dLN, as observed at day 7, and then left the dLN which partially emptied at day 21. One striking observation was the marked increase of AluDia particles in spleen at day 7 (54,500 particles) with a decrease at day 21 (7,000 particles). This massive alum access to spleen at day 7 was not previously noted in the Khan *et al.* study [3] which had no intermediate time points between day 4 and day 21. This observation is in keeping with the time frame of a primary immune response in the lymphoid organs. Particles were also detected in the liver, an organ not studied by Khan *et al.* [3], but previously shown to incorporate alum adjuvants from the circulation [31]. Besides additional insights provided by the evaluation of different time points and additional organs, the use of AluDia allowed us to substantiate our previous contention that alum particles translocate from the injected muscle to dLNs and then to distant organs unplugged to lymphatic vessels [3].

**Table 3:** AluDia particle systemic distribution at 7 and 21 days after injection of an equivalent of 400 μg/Kg aluminum in the tibialis anterior muscle. *AluDia* Alhydrogel^®^ and mfND complex, *dLNs* draining lymph nodes, *mfNDs* modified fluorescent nanodiamonds

Organs	mfND signals/organ at day 7	mfND signals/organ at day 21
dLNs	2552 ± 22	308± 16
Spleen	54507± 197	7000± 99
Liver	495 ± 84	2750± 56
Brain	Not done	15± 4

Four mice were used in each experimental point (n = 4 per organ and per time point). Results are mean ± SD

Four brains were examined at day 21 after i.m. injection of AluDia. Consistent with the low level of cerebral incorporation of particle previously noted at this early time point [3], each of the four brains contained 15 ± 4 AluDia particles, usually forming small clusters in the cerebellum or cerebral cortex. As shown in Fig. 3j-l, Morin stain for aluminum revealed that mfNDs and alum were colocalized in most particles, whereas occasional particles were solely positive for either Morin+ or fND+. Since the labeling of Alhydrogel^®^ with mfNDs confers physicochemical properties to the neo-particle that are very similar to whole vaccine particles, these data definitely establish that *bona fide* alum adjuvants of vaccines can penetrate in the brain [3]. This occurs in the particulate form and mimics brain translocation of infectious particles, such as intracellular bacteria, HIV and other pathogens [32–34].

Finally, AluDia particles confirmed the biodistribution modalities of poorly degradable particles. In addition, AluDia particles were well characterized whereas previously used AlRho particles had undetermined size, zeta potential, ultrastructure and proportion relative to alum. In addition to good relevance to vaccine, AluDia particles allowed administration of precise amounts of aluminum.

### *In vitro* observations

#### Cytotoxicity

**Fig. 4:**
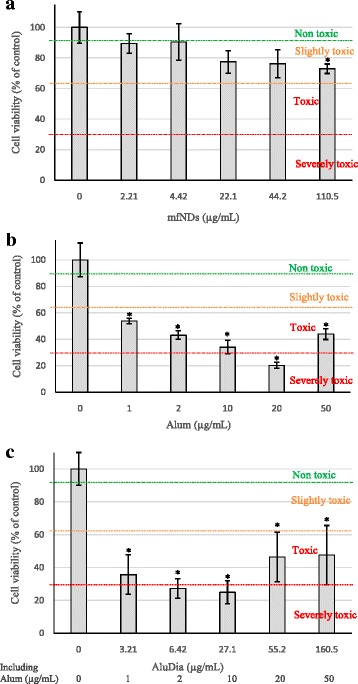
Cell viability assayed by mitochondrial metabolism assessment (MTT test). NSC-34 neuron-like cells were incubated with different concentrations of mfNDs, alum and AluDia particles for 72 hours. AluDia concentrations tested in (**c**) = mfNDs concentrations in (**a**) + alum concentrations in (**b**). **a**: mfNDs particles are non-toxic, except at the highest dose. **b**: alum particles display a toxic or severely toxic effect at all doses used. **c**: AluDia particles have no supplemental toxicity compared to alum alone. Viability was normalized to the value determined in untreated cells. Results are expressed as mean ± standard deviation. Cells were obtained from four different cultures to realize four biological replications (n = 4). Viability measurement of each concentration point was repeated 12 times. *Significant difference at p < 0.05. *AluDia* Alhydrogel^®^ and mfND complex, *mfNDs* modified fluorescent nanodiamonds

Since little has been reported about toxic effects of alum *in vitro*, we examined next whether AluDia particles could be used *in vitro* to study what could be the impact of alum on cultured cells. In order to compare the cytotoxicity of alum and AluDia particles, we incubated NSC-34 neuronal lineage cells with different concentrations of mfNDs, Alhydrogel^®^, and AluDia particles for 72 hours. Particle toxicity was evaluated based on cell viability assessed by MTT assay relative to controls, as proposed by Kong *et al.* [35]: (1) non-toxic >90 % cell viability; (2) slightly toxic = 65–90 %; (3) toxic = 35–65 %; (4) severely toxic ≤35 %. mfNDs appeared non-toxic, except at the highest dose (Fig. 4a), confirming previous reports on the lack of toxicity of nanodiamonds [36, 37]. Paget *et al*. showed that mfNDs are neither cytotoxic nor genotoxic on six human cell lines representative of potential target organs: HepG2 and Hep3B (liver), Caki-1 and Hek-293 (kidney), HT29 (intestine) and A549 (lung) [38]. These authors did not check mfNDs cytotoxicity on neural cell lines, but Hsu *et al.* reported that mfNDs disturb neither neuronal differentiation nor neuron functions [14]. In addition, mfNDs were shown to be non-toxic *in vivo,* in both *Caenorhabditis elegans* and mouse [39, 40]. However, other studies showed slight toxic and genotoxic effects of nanodiamonds *in vitro* and *in vivo* [41–43]. Only a few studies reported serious toxic effects *in vivo* [44, 45].

In contrast to mfNDs, alum particles were toxic or severely toxic at all doses used (Fig. 4b), and the same was observed with AluDia (Fig. 4c). AluDia had no supplemental toxicity compared to Alhydrogel^®^ particles alone. Interestingly, with both Alhydrogel^®^ and AluDia, cytotoxity did not show a linear dose–response, since higher doses tended to be less toxic than intermediate doses, as previously noted for particle toxicity [46–49]. Alhydrogel^®^*in vitro* toxicity for neuronal cells is in keeping with mouse studies showing *in vivo* neurotoxic effects of subcutaneously administered Alhydrogel^®^, including neural apoptosis and both motor and behavioral deficits [50].

#### Ultrastructural studies

**Fig. 5:**
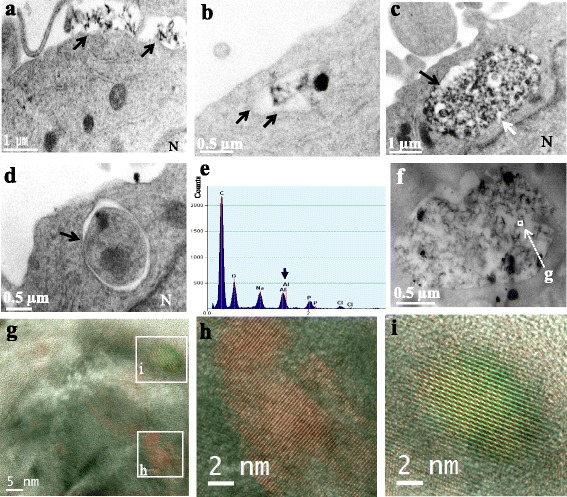
Ultra-structure observations by TEM of AluDia interaction with THP-1 monocyte cell line. Cells were treated with 20 μg/mL of AluDia particles for 3 (**a**) or 24h (b-i). **a** and **b**: AluDia particle internalization by THP-1 cells. Arrows indicate endosome membranes. **c**: AluDia particles inside macropinosome. Black (**b,c**) and white (**c**) arrows indicate macropinosome membrane and its absence, respectively. **d**: Intracellular AluDia particles encircled by double membrane autophagosome (arrow) indicating the autophagy activation. **e**: Detection of aluminum specific emission peak of (**h**) region, of internalized AluDia, by the X-ray microanalysis (EDX). **f**: macropinosome filled with AluDia particles. **g- i**: High resolution TEM analysis of the endosome content identified the specific crystal periodicity of mfNDs, (**h**): red (mfNDs) and green (alum) pseudo-colors, are superimposed to show the two crystalline structures of the AluDia complex (yellow pseudo-color, **i**). *AluDia* Alhydrogel^®^ and mfND complex, *mfNDs* modified fluorescent nanodiamonds, *TEM* transmission electron microscopy

Electron microscopy was performed on THP-1 monocyte/macrophage lineage cells incubated with AluDia particles. Particles were internalized by THP-1 cells within hours (Fig. 5a and b). After 24 hours AluDia particles were often found in large intracellular structures that may suggest macropinosomes, as was reported by Alhaddad *et al.* for siRNA delivery by nanodiamonds [51]. However, macropinosomes filled with AluDia particles often have damaged membranes (Fig. 5c and f). This observation was in line with the toxicity of alum for membrane lipid bilayers [52–54]. It seems possible that alum crystals directly aggress membranes [55], and this may play a crucial role in its adjuvant effect by inducing lysosomal function blockade [52–55]. Another mechanism of endosomal membrane damage may be related to nanomaterial-induced oxidative stress [56], and, indeed, aluminum [57], but not nanodiamonds [58], induces significant oxidative stress.

Another ultrastructural finding after 24-hour AluDia exposure consisted in intracellular particles encircled by double membranes highly suggestive of autophagophores, thus assessing active autophagy (Fig. 5d). Cells coping with microbes use a dedicated form of autophagy termed “xenophagy” as a host defense mechanism to engulf and degrade intracellular pathogens. The same holds true for inert particles subjected to phagocytosis/endocytosis [59]. Eidi *et al.* reported that the free internalized particles in cell cytoplasm could induce stress of mitochondria or other intracellular organelles resulting in autophagy activation [56]. As mentioned above, alum particles are toxic to membranes which destabilizes phagosomes and lysosomes, triggers inflammasome assembly, and impedes the autophagy pathways [52–55]. It seems possible that macrophages that perceive the foreign particles in their cytosol, just like senescent organelles or bacteria, will attempt to reiterate the autophagic process until they dispose of the alien materials. The compartmentalization of particles within double membrane and subsequent fusion of autophagosomes with repaired and re-acidified lysosomes could expose alum particles to lysosomal acidic pH, a crucial factor in the alum solubilization process. Notably, Li *et al.* reported that alpha-alumina nanoparticles activate the autophagy in dendritic cells much more efficiently than alum particles [60]. To our knowledge, no study in the literature has reported autophagy activation by mfNDs.

It is difficult to visualize moderate amounts of alum within cells by TEM, and one has to use X-ray microanalysis (EDX) to assess the presence of aluminum by detection of a specific emission peak (Fig. 5e). This approach has limitations since the sample is always at risk of being contaminated by extrinsic aluminum present in the air or incorporated into the sample during its processing. The use of AluDia may help in tracking alum in resin embedded material. MfNDs cannot represent contaminants and their specific and highly photostable fluorescence can be detected in semi-thin sections. Moreover, high resolution TEM can reliably identify the specific crystalline structures. As shown in Fig. 5f-i, high resolution TEM of the endosome content of THP1 cells exposed to AluDia identified the specific crystal periodicity of both mfNDs (red pseudo-color) and Alhydrogel^®^ (green pseudo-color), as well as superimposition of the two crystalline structures (yellow pseudo-color). This confirmed the stability of AluDia after internalization by immune cells. Using mfNDs as a tag mimicing vaccine antigen, the same approach could be used to assess if and how the alum adjuvant dissociates from compounds adsorbed at its surface over a long time *in vivo*.

## Conclusion

We developed a tracking method for aluminum adjuvant particles to be able to understand their fate, residence time, putative accumulation and impact on organs and the organism based on their labeling with functionalized nanodiamonds. The detection of mfNDs by photoluminescence is easy to implement at different scales and allows a detailed estimation of biodistribution in organs and the organism down to the subcellular level. Adjuvant particle labeling with mfNDs affects neither their physicochemical characteristics nor their biological effects. Thus, fluorescent nanodiamonds modified by hyperbranched polyglycerol appear to be a biocompatible and original tool to address all aspects of alum biodisposition.

## Abbreviations

AlRho: Alum-rhodamine nanohybrid
AluDia: Alhydrogel^®^ and mfND complex
ASIA: Autoimmune/auto-inflammatory syndrome induced by adjuvant
dLNs: Draining lymph nodes
D-MEM: Dulbecco’s modified Eagle’s medium
EDX: Energy-dispersive x-ray
fNDs: Fluorescent nanodiamonds
HBG: hyperbranched polyglycerol
HBV: Hepatitis B virus
HIV: Human immunodeficiency virus
HR-TEM: High-resolution transmission electron microscope
mfNDs: Hyperbranched polyglycerol modified fluorescent nanodiamonds
MMF: Macrophagic myofasciitis
NV: Nitrogen vacancy centers
PBS: Phosphate buffered saline
PFA: Paraformaldehyde
STEM: Scanning transmission electron microscopy
TEM: Transmission electron microscope
